# ﻿Molecular and morphological evidence reveals a hidden new taxon in the endemic genus *Pseudocuneopsis* (Bivalvia, Unionidae) from China

**DOI:** 10.3897/zookeys.1179.109817

**Published:** 2023-09-11

**Authors:** Lili Liu, Liping Zhang, Dandong Jin, Haotian Wang, Xiongjun Liu, Ruiwen Wu

**Affiliations:** 1 School of Life Science, Shanxi Normal University, Taiyuan 030031, China Shanxi Normal University Taiyuan China; 2 Datian High School, Linhai 317004, China Datian High School Linhai China; 3 China Development Bank Guangxi Zhuang Autonomous Region Branch, Nanning 530025, China China Development Bank Guangxi Zhuang Autonomous Region Branch Nanning China; 4 School of Life Science, Jiaying University, Meizhou 514015, China Jiaying University Meizhou China

**Keywords:** Bivalves, COI, freshwater mussel, morphology, taxonomy

## Abstract

A new species of freshwater mussel belonging to the genus *Pseudocuneopsis*, namely *Pseudocuneopsiswuana***sp. nov.**, is diagnosed and described from Guangxi Province, China. This paper provides a detailed shell morphological description, soft-body anatomical characteristics, and partial sequences of mitochondrial COI as DNA barcode data for the novel species. The new species can be distinguished from its congeners (*Pseudocuneopsissichuanensis*, *P.yangshuoensis*, and *P.capitata*) by shell shape, beak position, and surface sculpture. Phylogenetic analyses based on the mitochondrial COI gene reveal that *Pseudocuneopsiswuana***sp. nov.** forms a sister group with *P.yangshuoensis* and exhibits an interspecific genetic distance of 5.1%. Therefore, we provide robust morphological and molecular evidence to support the validity of this new species.

## ﻿Introduction

The Unionidae Gray, 1840 is a family of freshwater bivalves (Mollusca, Bivalvia, Unionida) commonly known as freshwater mussels (Lopes-Lima et al. 2014; Graf and Cummings 2021). These bivalves are important components of freshwater ecosystems, providing various ecosystem services such as nutrient cycling, water purification enhancement, bioturbation, and habitat creation (Vaughn 2018).

China is widely recognized as one of the major biodiversity hotspots for freshwater mussels owing to its abundant rivers and lakes which harbor a wealth of endemic species (Zieritz et al. 2018; Liu et al. 2022). However, field investigations of and research on unionids have been primarily focused on the middle and lower reaches of the Yangtze River (e.g. Wu et al. 2018; Huang et al. 2019; Liu et al. 2020, 2022), with less sampling in other river basins in southwest China, such as the river systems of Guangxi Province. These under-investigated areas severely limit our ability to discover new species and hinder a comprehensive understanding of phylogeny and evolution within this group.

The genus *Pseudocuneopsis* Huang, Dai, Chen & Wu, 2022 was recently established by Wu et al. (2022). Based on mitochondrial phylogenomic analyses, Wu et al. (2022) confirmed that the genus *Cuneopsissensu lato* was polyphyletic and proposed two new genera: *Arcuneopsis*Wu et al., 2022 and *Pseudocuneopsis*Wu et al., 2022. However, *Arcuneopsis* was later considered a junior objective synonym of *Tchangsinaia* Starobogatov, 1970 because these genera have the same type species, *Uniopisciculus* Heude, 1874 (Starobogatov 1970). Currently, comprehensive molecular systematics have stabilized the taxonomic status of *Pseudocuneopsis* as a member of the subfamily Unioninae in the Unionidae (Huang et al. 2019; Wu et al. 2019; Wu et al. 2022). The genus comprises three recognized species endemic to China (Graf and Cummings 2023; MolluscaBase eds 2023): *Pseudocuneopsissichuanensis* Huang, Dai, Chen & Wu, 2022; *P.capitata* (Heude, 1874); and *P.yangshuoensis* Wu & Liu, 2023. While *P.sichuanensis* has a narrow distribution and is reported only from the Sichuan Province; *P.capitata* is widely distributed throughout the Yangtze river basin (Liu et al. 1979; Liu et al. 2022; Wu et al. 2022). *Pseudocuneopsisyangshuoensis* is the recently discovered and described addition to this group from Guangxi, as reported by us (Wu et al. 2023).

In this study, another new species of *Pseudocuneopsis* from Guangxi is diagnosed and described. In addition, we provide estimations of the intraspecific and interspecific genetic distances within *Pseudocuneopsis* based on the mitochondrial COI barcode fragment to examine this species’ validity.

## ﻿Materials and methods

### ﻿Specimen collection, identification, and anatomical observations

In June 2023, six samples with tissues were collected from the Qingshui River, Nanning City, Guangxi Province, China (23.4075°N, 108.7557°E). All specimens are deposited as vouchers at the Museum of Zoology, Shanxi Normal University (**SXNU**), China (voucher numbers SXNU23062201–SXNU23062206). We performed dissections on all individuals to observe the soft-body characteristics through visual examination by eye and through a stereoscopic microscope.

### ﻿DNA extraction and COI amplification

Total genomic DNA was extracted from dissected somatic tissues using TIANamp Marine Animals DNA Kit (Tiangen Biotech, Beijing, China) according to the manufacturer’s instructions.

Polymerase chain reaction (PCR) amplification of the COI gene with a 680-base pair fragment was performed using a primer pair consisting of (LCO22me2 + HCO700dy2) (Walker et al. 2007). Thermal cycling conditions were 98 °C for 10 s, followed by 35 cycles of 94 °C for 1 min, 50 °C for 1 min, 72 °C for 1–2 min, and a final extension of 72 °C for 7 min, following the TaKaRa Ex manufacturer’s protocol. The amplified PCR products were purified and sequenced by Sangon Biotech (Shanghai). The sequences obtained in this study have been uploaded to GenBank (OR297986–OR297991).

### ﻿DNA barcode dataset construction

We compiled a mitochondrial COI dataset by incorporating newly obtained sequences from this study and available sequences of *Pseudocuneopsissichuanensis*, *P.yangshuoensis*, and *P.capitata* from GenBank. Additionally, we downloaded GenBank COI sequences of 30 species of the subfamily Unioninae as the ingroup and two species of the subfamily Gonideinae as the outgroup to augment our dataset.

Finally, our study used a total of 38 COI sequences; detailed sequence information and GenBank accession numbers are provided in Table 1.

**Table 1.** List of COI sequences used in this study.

Taxa	GenBank accession number
UNIONINAE Rafinesque, 1820	
*Lasmigonacompressa* (Lea, 1829)	AF156503
*Pyganodongrandis* (Say, 1829)	AF231734
*Strophitusundulatus* (Say, 1817)	AF156505
*Pseudanodontacomplanata* (Rossmässler, 1835)	KX822661
*Uniotumidus* (Philipsson, 1788)	KX822672
*Nodulariadouglasiae* (Griffith & Pidgeon, 1833)	NC_026111
*Aculamprotulascripta* (Heude, 1875)	MF991456
*Aculamprotulatientsinensis* (Crosse & Debeaux, 1863)	NC_029210
*Acuticostachinensis* (Lea, 1868)	MG462919
*Cuneopsisheudei* (Heude, 1874)	MG462974
*Cuneopsisrufescens* (Heude, 1874)	MG462982
*Inversiunioyanagawensis* (Kondo, 1982)	LC518988
*Pseudocuneopsiscapitata* (Heude, 1874)	MZ540968
*Pseudocuneopsiscapitata* (Heude, 1874)	MZ540969
*Pseudocuneopsissichuanensis* Huang, Dai, Chen & Wu, 2022	MZ540966
*Pseudocuneopsissichuanensis* Huang, Dai, Chen & Wu, 2022	MZ540967
*Pseudocuneopsisyangshuoensis* Wu & Liu, 2023	OQ696218
*Pseudocuneopsisyangshuoensis* Wu & Liu, 2023	OQ696219
*Pseudocuneopsisyangshuoensis* Wu & Liu, 2023	OQ696220
*Pseudocuneopsisyangshuoensis* Wu & Liu, 2023	OQ696221
*Pseudocuneopsisyangshuoensis* Wu & Liu, 2023	OQ696222
*Pseudocuneopsiswuana***sp. nov.** 1*	OR297986
*Pseudocuneopsiswuana***sp. nov.** 2*	OR297987
*Pseudocuneopsiswuana***sp. nov.** 3*	OR297988
*Pseudocuneopsiswuana***sp. nov.** 4*	OR297989
*Pseudocuneopsiswuana***sp. nov.** 5*	OR297990
*Pseudocuneopsiswuana***sp. nov.** 6*	OR297991
*Tchangsinaiapiscicula* (Heude, 1874)	KJ434496
*Tchangsinaiapiscicula* (Heude, 1874)	KJ434497
*Tchangsinaiapiscicula* (Heude, 1874)	KJ434498
*Tchangsinaiapiscicula* (Heude, 1874)	KJ434499
*Schistodesmuslampreyanus* (Baird & Adams, 1867)	MG463038
*Schistodesmusspinosus* (Simpson, 1900)	MG463045
*Lanceolariagladiola* (Heude, 1877)	KY067441
*Lanceolariagrayii* (Griffith & Pidgeon, 1833)	NC_026686
*Lanceolarialanceolata* (Lea, 1856)	NC_023955
GONIDEINAE Ortmann, 1916	
*Lamprotulaleaii* (Gray, 1833)	NC_023346
*Sinosolenaiaoleivora* (Heude, 1877)	KX822670

* Generated in this study.

COI nucleotide sequences were aligned under the invertebrate mitochondrial code mode in MACSE (Ranwez et al. 2021) with default settings. We calculated and compared inter- and intraspecific distances with MEGA 7.0 (Kumar et al. 2016) using the uncorrected *p*-distance. Standard error was assessed using 1000 bootstrap replicates.

### ﻿Phylogenetic analysis

Bayesian-inference (BI) analyses were inferred in MrBayes (Ronquist et al. 2012), using the GTR+I+G model of nucleotide substitution. Four chains were run simultaneously for 10 million generations and trees were sampled every 1000 generations. The first 25% of these trees were discarded as burn-in when computing the consensus tree (50% majority rule). Sufficient mixing of the chains was considered to have been reached when the average standard deviation of split frequencies was below 0.01. Additionally, IQ-TREE was run for maximum-likelihood (ML) tree reconstruction with 1000 ultrafast bootstrap replications (Minh et al. 2013).

## ﻿Taxonomy

### ﻿ Pseudocuneopsis wuana

Taxon classificationAnimaliaUnionidaUnionidae

Liu & Wu sp. nov.

DB206623-4B27-5A04-88FC-19D28DDE575E

https://zoobank.org/7D3D0EF8-1231-4610-AE5B-07E9A64E3F86

Fig. 1

#### Type specimens.

***Holotype***: China • Guangxi Province, Nanning City (南宁市), Qingshui River (23.4075°N, 108.7557°E), 22 June 2023, Ruiwen Wu leg. (SXNU23062201). ***Paratypes***: same data as holotype (voucher numbers SXNU23062202–SXNU23062206).

**Figure 1.**
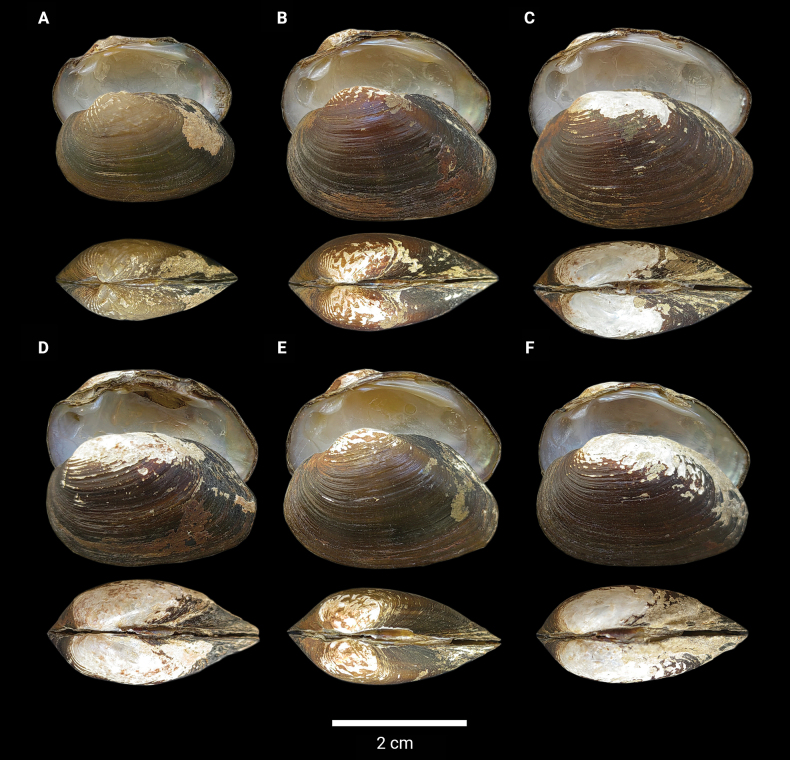
Photographs of *Pseudocuneopsiswuana* sp. nov. **A** holotype (SXNU23062201) **B–F** paratypes (SXNU23062202–SXNU23062206). All specimens shown at the same scale. Photographs by Lili Liu and Liping Zhang.

#### Diagnosis.

*Pseudocuneopsiswuana* sp. nov. is morphologically distinct from the other three recognized species within the genus by shell shape, beak position and sculpture, and surface sculpture (Table 2). Diagnostic characteristics: shell ovaliform; ventral margin somewhat prominent at middle; umbo situated 1/3–1/4 of shell length and higher than dorsal margin; epidermis tawny to dark brown, shell surface sculptured with concentric ridges; nacre silvery-white, umbo pocket light yellow.

**Table 2.** Conchological characters of *Pseudocuneopsiswuana* sp. nov., *P.yangshuoensis*, *P.capitata*, and *P.sichuanensis*. Characteristic descriptions of *P.capitata*, *P.sichuanensis*, and *P.yangshuoensis* are referenced from published works (Wu et al. 2022; Wu et al. 2023).

	*P.yangshuoensis*	*P.sichuanensis*	*P.capitata*	*P.wuana* sp. nov.
**Length**	41.39–50.51 (mm)	49.16–62.97 (mm)	101.68–121.32 (mm)	24.97–35.91 (mm)
**Width**	15.34–19.40 (mm)	15.01–22.42 (mm)	37.07–42.72 (mm)	10.72–15.74 (mm)
**Height**	27.25–28.99 (mm)	27.16–36.02 (mm)	49.23–61.02 (mm)	15.49–21.95 (mm)
**Shell shape**	Wedge-shaped	Oval	Elongate	ovaliform
**Umbo position**	1/3 of shell length; umbo obviously lower than the dorsal margin	1/4–1/5 of shell length; umbo slightly higher than the dorsal margin	1/6 of shell length; umbo obviously higher than the dorsal margin	1/3–1/4 of shell length; umbo higher than the dorsal margin
**Surface sculpture**	Epidermis brownish-black covered with concentric ridges	Epidermis dark brown with growth annuli with 1 or 2 sulci near posterior dorsal margin	Epidermis brownish with low rides, which follow growth lines	Epidermis tawny to dark brown covered with concentric ridges
**Nacre colou**r	Orange	White	Milk-white	Silvery-white, umbo pocket light yellow
**Dorsal margin**	Anterior margin oval, and inflated, with the dorsal margin curved downwards	Anterior margin oval, and inflated, with dorsal margin curved downwards	Anterior margin oval, highly inflated, dorsal margin sloped downwards	Anterior margin round, and inflated, with dorsal margin curved downwards
**Posterior slope**	Blunt	Blunt	Sharp	Blunt
**Ventral margin**	Nearly straight or slightly concave	Slightly concave inward at middle posterior	Rounded anteriorly, with sinus behind anterior inflation	Somewhat prominent at middle

#### Description.

Shell ovaliform, medium-thick; anterior margin rounded and inflated; ventral margin somewhat prominent in the middle; umbo located at 1/3–1/4 of shell length and higher than dorsal margin; umbo sculptured with nodes or nodulose wrinkles, or severely eroded; posterior slope formed by ventral margin and dorsal margin low, blunt, located at almost 1/3 of shell height; epidermis tawny to dark brown covered with concentric ridges; anterior adductor muscle scars elliptical, deep, and smooth; posterior adductor muscle scars round to elliptical, shallow and smooth; anterior and posterior retractor muscle scars noticeable, with anterior and posterior irregularly oval; mantle muscle scars obvious; left valve with two separate pseudocardinal teeth and two lateral teeth; outer and inner pseudocardinal teeth of different lengths and projecting outward at different levels, outer and inner pseudocardinal teeth roughly the same size; right valve with one well-developed pseudocardinal tooth and one lateral tooth; nacre silvery-white, umbo pocket light yellow.

Length 24.97–35.91 mm, height 15.49–21.95 mm.

#### Etymology.

This species’ name is dedicated to Dr Ruiwen Wu, who collected these specimens. For the common name, we recommend “Wu ovaliform Mussel” (English) and “Wu Shi Wei Xie Bang” (武氏伪楔蚌) (Chinese).

#### Distribution.

**Figure 2.**
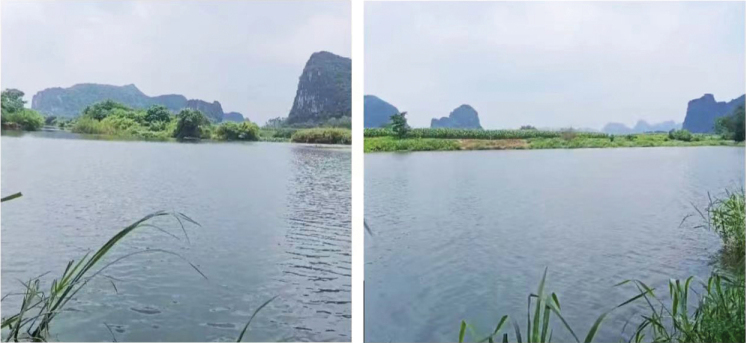
The type locality of *Pseudocuneopsiswuana* sp. nov. in Qingshui River, Nanning City, China.

Qingshui River, Guangxi, China (Fig. 2).

#### Anatomical characteristics.

**Figure 3.**
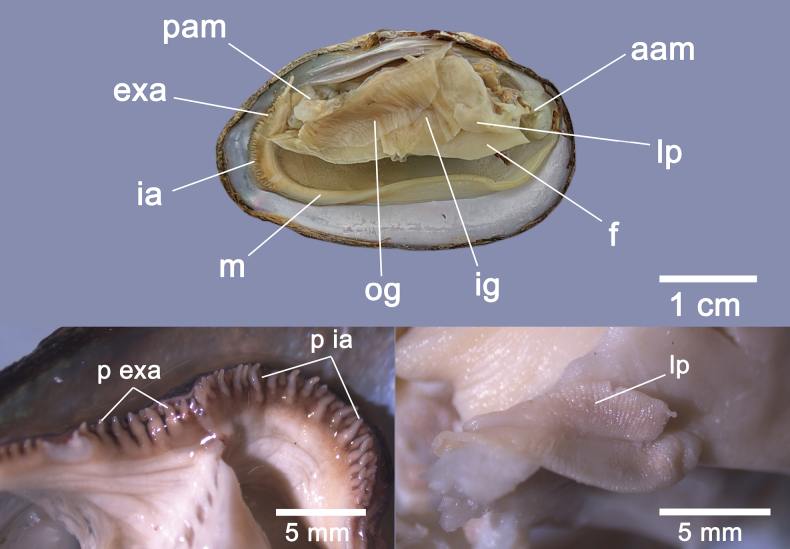
Anatomical features of *Pseudocuneopsiswuana* sp. nov. with right valve removed. Abbreviations: aam, anterior adductor muscle; pam, posterior adductor muscle; exa, excurrent aperture; ia, incurrent aperture; f, foot; ig, inner gill; og, outer gill; lp, labial palps; m, mantle; p ia, papillae in incurrent aperture; p exa, papillae in excurrent aperture.

Within the incurrent aperture, there are elongated papillae arranged in three or four rows; these have a slight swelling at their base; papillae of the excurrent aperture well developed, stubby, and arranged in two rows. The inner gills are larger than the outer gills. Labial palps are medium-thick and elongated (Fig. 3).

#### Molecular analyses.

Pairwise COI sequence divergences from *Pseudocuneopsiswuana* sp. nov., *P.yangshuoensis*, *P.capitata*, and *P.sichuanensis* were calculated in MEGA 7.0 with the uncorrected *p*-distance model. The intraspecific divergence of the newly discovered species, *P.wuana* sp. nov., ranged from 0% to 0.5%. The genetic divergence between *P.wuana* and *P.yangshuoensis* was 5.1%, while that between *P.wuana* and the other two species, namely *P.sichuanensis* and *P.capitata*, was 8.2% and 10.2%, respectively.

**Figure 4.**
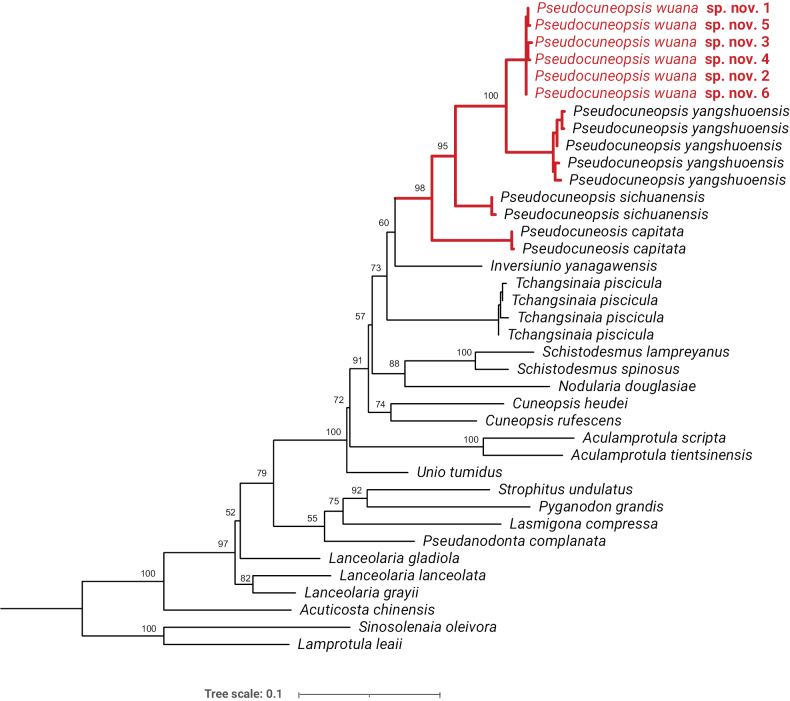
Phylogenetic tree of freshwater mussels inferred by maximum-likelihood (ML) analysis of the COI barcode fragment. Bootstrap-support (BS) values are shown at the nodes. The new species is indicated in red.

**Figure 5.**
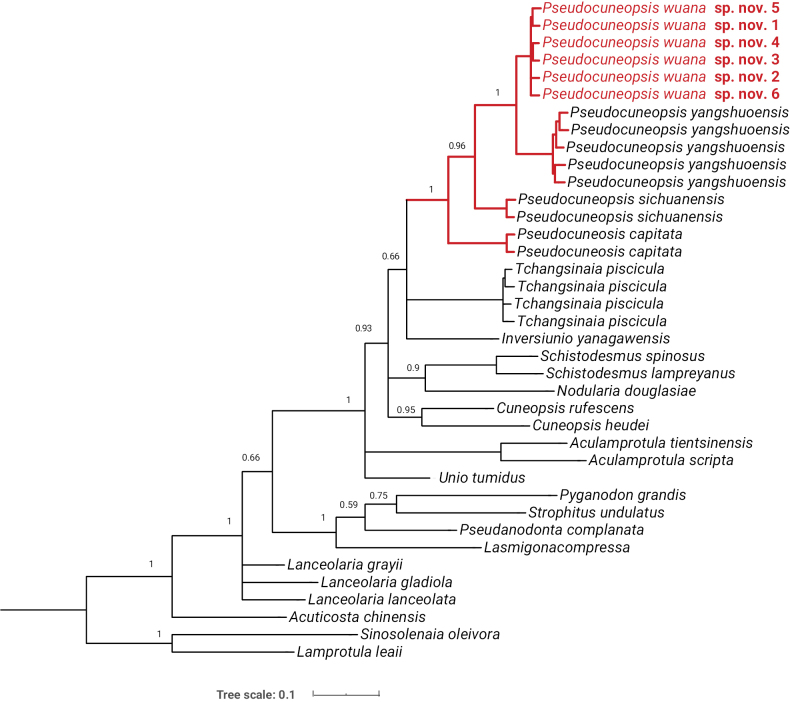
Phylogenetic tree of freshwater mussels inferred from Bayesian-inference (BI) analysis of the COI barcode fragment. Posterior probabilities (PP) are shown at the nodes. The new species is indicated in red.

The BI and ML trees based on the mitochondrial COI gene yielded incongruent topologies (Figs 4, 5). However, both phylogenetic trees consistently supported the sister-group relationship between *Pseudocuneopsiswuana* sp. nov. and *P.yangshuoensis* (BS = 100%; PP = 1.00, Figs 4, 5). The genus *Pseudocuneopsis* exhibited monophyly with robust bootstrap support (BS = 98%) and full Bayesian posterior probability (PP = 1.00) (Figs 4, 5).

#### Remarks.

The placement of the new species in *Pseudocuneopsis* is supported by both morphological characteristics and phylogenetic analyses. *Pseudocuneopsiswuana* sp. nov. can readily be distinguished from congeneric species by its distinctive ovaliform shell, tawny to dark-brown epidermis covered with concentric ridges, and somewhat prominent at the middle of ventral margin. We conducted an analysis of interspecific divergence among *P.sichuanensis*, *P.capitata*, *P.yangshuoensis*, and the newly described species *P.wuana* using the COI gene. The results indicate that the genetic distances between *P.wuana* and its congeneric species, namely *P.yangshuoensis*, *P.sichuanensis*, and *P.capitata*, were 5.1%, 8.2%, and 10.2%, respectively, which are distinctly higher than intraspecific divergences. We conclude that the genetic analyses support the recognition of *P.wuana* sp. nov. as a valid new species, which can easily be distinguished by its unique COI barcode sequences.

In recent years, through more extensive investigations and field surveys, several new species of freshwater mussels, such as *Postolataguangxiensis* and *Pseudocuneopsisyangshuoensis*, have been discovered in Guangxi Province, China (Dai et al. 2023; Wu et al. 2023). As a major ecological barrier in southwest China, Guangxi is one of the significant watershed areas of the Pearl river basin. The province is noted for its distinctive karst landscape which harbors a rich diversity of species. However, due to the insufficient attention paid to freshwater mussels in the region, the mussel diversity in China is poorly understood. The lack of data regarding species’ distributions, population trends, threats, and accurate taxonomic information has severely impeded conservation efforts for the unionids in this area. Our findings suggest that there is still much to be discovered regarding the diversity of freshwater mussels in Guangxi Province. Additional extensive exploration may reveal other species that have yet to be documented.

## Supplementary Material

XML Treatment for Pseudocuneopsis wuana

## ﻿Additional information

### Author ORCIDs

Lili Liu https://orcid.org/0009-0009-1164-7735

Xiongjun Liu https://orcid.org/0000-0002-5333-6670

Ruiwen Wu https://orcid.org/0000-0002-8936-6054

### Data availability

All of the data that support the findings of this study are available in the main text.
